# Changing trends of birth weight with maternal age: a cross-sectional study in Xi’an city of Northwestern China

**DOI:** 10.1186/s12884-020-03445-2

**Published:** 2020-11-30

**Authors:** Shanshan Wang, Liren Yang, Li Shang, Wenfang Yang, Cuifang Qi, Liyan Huang, Guilan Xie, Ruiqi Wang, Mei Chun Chung

**Affiliations:** 1 Department of Obstetrics and Gynecology, Maternal & Child Health Center, The First Affiliated Hospital of Xi’an Jiaotong University, No. 277, West Yanta Road, 710061 Xi’an, Shaanxi People’s Republic of China; 2 Department of Obstetrics and Gynecology, Xijing Hospital, the Fourth Military Medical University, Shaanxi Xi’an, PR China; 3 School of Public Health, Xi’an Jiaotong University Health Science Center, Shaanxi Xi’an, PR China; 4 Department of Public Health and Community Medicine, Tufts University School of Medicine, Massachusetts Boston, USA

**Keywords:** Maternal age, Birth weight, Low birth weight, Macrosomia

## Abstract

**Background:**

Most studies have shown that maternal age is associated with birth weight. However, the specific relationship between each additional year of maternal age and birth weight remains unclear. The study aimed to analyze the specific association between maternal age and birth weight.

**Methods:**

Raw data for all live births from 2015 to 2018 were obtained from the Medical Birth Registry of Xi’an, China. A total of 490,143 mother-child pairs with full-term singleton live births and the maternal age ranging from 20 to 40 years old were included in our study. Birth weight, gestational age, neonatal birth date, maternal birth date, residence and ethnicity were collected. Generalized additive model and two-piece wise linear regression model were used to analyze the specific relationships between maternal age and birth weight, risk of low birth weight, and risk of macrosomia.

**Results:**

The relationships between maternal age and birth weight, risk of low birth weight, and risk of macrosomia were nonlinear. Birth weight increased 16.204 g per year when maternal age was less than 24 years old (95%CI: 14.323, 18.086), and increased 12.051 g per year when maternal age ranged from 24 to 34 years old (95%CI: 11.609, 12.493), then decreased 0.824 g per year (95% CI: -3.112, 1.464). The risk of low birth weight decreased with the increase of maternal age until 36 years old (OR = 0.917, 95%CI: 0.903, 0.932 when maternal age was younger than 27 years old; OR = 0.965, 95%CI: 0.955, 0.976 when maternal age ranged from 27 to 36 years old), then increased when maternal age was older than 36 years old (OR = 1.133, 95%CI: 1.026, 1.250). The risk of macrosomia increased with the increase of maternal age (OR = 1.102, 95%CI: 1.075, 1.129 when maternal age was younger than 24 years old; OR = 1.065, 95%CI: 1.060, 1.071 when maternal age ranged from 24 to 33 years old; OR = 1.029, 95%CI: 1.012, 1.046 when maternal age was older than 33 years old).

**Conclusions:**

For women of childbearing age (20–40 years old), the threshold of maternal age on low birth weight was 36 years old, and the risk of macrosomia increased with the increase of maternal age.

## Background

Birth weight (BW) is the most important index reflecting intrauterine growth and development of newborns, and is also a vital index to evaluate the health status of the newborns [1]. Abnormal BW, including low birth weight (LBW, BW < 2500 g) and macrosomia (BW ≥ 4000 g), significantly increases the risk of perinatal mortality and morbidity and, in recent years, has been shown to be a marker of age-related disease risk [2, 3]. As the most common adverse birth outcome, the incidence of abnormal BW is generally high in the world. It was estimated that the incidence of LBW was about 5–7% in developed countries and as high as 19% in developing countries [4], and in mainland China, the specific interest for this paper, it was 6.1% [5]. Furthermore, the incidence of macrosomia also has increased over the past two to three decades in both developed and developing countries, the prevalence of macrosomia was 9.2% in the United States and 7.3% in China recently [6, 7]. Considering the large number of newborns in China, it is likely that LBW and macrosomia might remain a major public health issue over the next few years in China [3].

With the development of society and change of people’s conception of procreation (assisted reproductive technology, the increased education and economic activities of women, reduced marriage rates and increased double income no kids), it was reported that the global trend of delayed childbirth is increasing, e.g. United States and Korea [8, 9]. Similarly in China, the mean maternal age and the proportion of elderly pregnant women(≥ 35 years) are increasing [10]. In 2011, a survey of 14 provinces in China showed that the average delivery age of pregnant women was 28 ± 5 years, the proportion of maternal age older than 35 was 7.8%, and the proportion of maternal age younger than 20 years old was 1.4% [6, 11]. The high proportion of pregnant women older than 35 years old is accompanied by the increased risk of miscarriage and chromosomal aneuploidy [12].

Previous studies indicated that the relationship between maternal age and BW was inconsistent [12–16]. Most of the previous studies focused on the incidence of LWB and macrosomia in different age groups by fixed classification of maternal age [13, 16]. This classification method with 35 years old as the boundary cannot accurately reflect the trend of BW and age, as this may underestimate the age related risk of younger age groups and overestimate risks in older age groups [17]. There is no sufficient evidence to assess the appropriateness of using this artificial traditional age classification to evaluate their impacts on BW. Further, with the increase of delayed childbearing in recent years, the development of medical technology and social and economic development, the current traditional methods of dividing age threshold may not be suitable for today’s situation. Especially for China, a country with a large birth population and a huge social change, this research is helpful to clinical decisionmaking and for public health policies. The objective of this study was to estimate the changes of BW according to 1-year intervals of maternal age among women, treating age as a continuous variable with a flexible, generalized additive approach.

## Methods

### ***Data Source and Study Population***

A cross-sectional study was conducted to determine the relationship between maternal age and BW with dose-response analyses. The data were obtained from the Birth Registry Database in Xi’an city, which covered all midwifery clinics and hospitals in the city. Dedicated trained health workers are responsible for collecting and registering birth information at each hospital. Validity was confirmed at various levels: by the Chief Midwife, the Chief Physician, and the Department of Medical Administration at each hospital; by the newborns’ parents at the time when the birth certificate was issued; and reviewed by issuing personnel. This analysis included all full-term singleton live births born from January 2015 to December 2018 in Xi’an city of Shaanxi province in China. We collected the information on maternal and newborn, including the number of births, BW, gestational age, neonatal birth date, maternal residence, maternal birth date, residence and ethnicity.

**Fig. 1:**
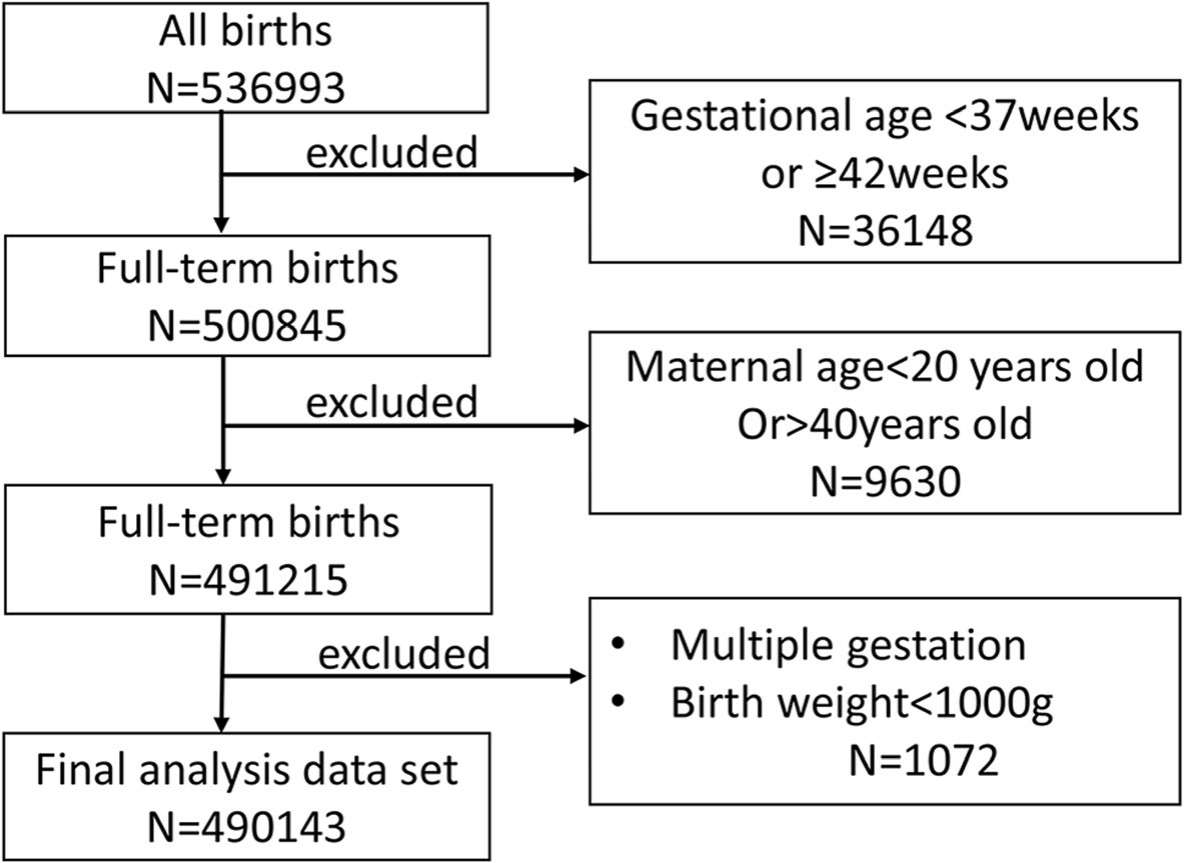
Flow chart of including criteria.

A total number of 536,993 mother-child pairs were abstracted from the Medical Birth Registry of Xi’an, born from Jan 2015 to Dec 2018. After removing those failing to meet the requirements explained below and unreasonable records, 490,143 mother-child pairs (91.3%) were included in the analysis. Newborns who were term birth (gestational age was between 37 to 41^+ 6^ weeks), singleton live births, and whose BW was greater than or equal 1000 g and maternal age at birth ranged from 20 to 40 years old were selected. Clinically, those extreme values (BW < 1000 g) were mainly caused by some clinical diseases rather than maternal age. These extremes can create biases that affect the exploration for real relationships between mother’s age and BW. Similarly, pregnant women over the age of 40 and under the age of 20 had more clinical complications and has too many confounding factors, which would cause biases. To get accurate results, we excluded this part of data. The flow chart of including criteria can be seen in Fig. 1.

The study protocol was approved by the Medical Ethics Committee of the First Affiliated Hospital of Xi’an Jiaotong University (No.XJTU1AF2018LSK-245). Data used in the study were all anonymous, and no individual identifiable information was available for the analysis. In the process of this study, all data were only used to conduct research, not for other purposes, which can ensure that the privacy of patients was fully protected.

### Measurements and Definitions of LBW and Macrosomia

The maternal age was analyzed as a continuous variable. BW (in grams) was measured routinely by registered midwives using an electronic weighting scale within 2 hours of delivery. The LBW was defined as BW less than 2500 g [18], and the macrosomia was defined as BW greater than or equal to 4000 g [19]. The season of birth was divided into spring, summer, autumn and winter according to the birth time. According to the Chinese lunar calendar, the winter, spring, summer and fall begin on December 1st, March 1st, June 1st and September 1st, respectively. The maternal residence was divided into 3 groups (urban, suburb, countryside). Maternal ethnicity was divided into 2 groups (Han and other).

### Statistical Analysis

We described the distribution of each group with the number and proportion of births. The maternal age at birth obeyed normal distribution after testing and was expressed as means ± standard deviation (means ± SD). The women’s characteristics was analyzed as categorical variables to examine the association with BW by one-way ANOVA and risk of LBW and macrosomia by Chi-square Test.

Generalized additive model (GAM) was conducted to see whether there were nonlinear relationships between maternal age and BW, LBW and macrosomia, respectively. And the relationships were further verified by adjusting the potential confounders found by the One-way ANOVA and Chi-square Test.

Two-piece wise linear regression model was used to examine the threshold effect of the maternal age on BW and the risk of LBW and macrosomia with an adjustment for potential confounders [20]. The turning point of maternal age was defined as where the relationships between maternal age and BW and the risk of LBW and macrosomia started to change. 푃<0.05 was considered as statistically significant.

All analyses were conducted with the statistical packages R (R Foundation; http://www.r-project.org; version 3.6.3) and Empower Stats (www.empowerstats.com; X&Y Solutions Inc).

## Results

### Related factors of the mother-child pairs

A total of 490,143 mother-child pairs were abstracted. The mean BW of newborns was 3364.937 ± 420.528 g, and the mean age of mothers was 28.728 ± 4.134 years old. The incidence of LBW was 1.5%, and the incidence of macrosomia was 6.0%. The basic characteristics of the mother-child pairs were shown in Table 1. There were significant differences in BW, the proportion of LBW and macrosomia among different gestational age groups, seasonal of birth groups, and maternal residence groups (P < 0.05), but not maternal ethnicity groups (*P* > 0.05).

**Table 1:** Basic characteristics of the mother-child pairs

Variables	Birth weight(*n* = 490,143)			Low birth weight(*n* = 7146)		Macrosomia(*n* = 29,457)	
	*N* (%)	Mean ± SD(g)	*F*	N(%)	*χ2*	N(%)	*χ2*
**Gestational age(weeks)**			51543.00*		15294.00*		7197.40*
37–37^+ 6^	36,138(7.4)	3020.404 ± 422.075		3125(8.6)		498(1.4)	
38–38^+ 6^	98,636 (20.1)	3247.870 ± 396.350		1994(2.0)		2946(3.0)	
39–39^+ 6^	162,966 (33.2)	3369.093 ± 394.169		1312(0.8)		8442(5.2)	
40–40^+ 6^	138,209 (28.2)	3462.910 ± 400.804		590(0.4)		11,373(8.2)	
41–41^+ 6^	54,194(11.1)	3545.406 ± 395.768		125(0.2)		6198(11.4)	
**Season of birth**			24.01*		13.97*		36.51*
Spring	119,481(24.4)	3363.061 ± 422.333		1806(1.5)		7137(5.9)	
Summer	121,425(24.8)	3360.024 ± 18.322		1859(1.5)		6932(5.7)	
Autumn	128,548(26.2)	3367.011 ± 419.182		1773(1.4)		7806(6.1)	
Winter	120,689(24.6)	3369.533 ± 422.324		1708(1.4)		7582(6.3)	
**Maternal residence**			243.76*		58.42*		40.42*
Urban	245,250(50.1)	3372.876 ± 416.985		3370(1.4)		15,193(6.2)	
Suburb	128,008(26.1)	3363.916 ± 422.330		1800(1.4)		7648(6.0)	
Countryside	116,885(23.8)	3349.401 ± 425.485		1976(1.7)		6616(5.7)	
**Ethnicity**			0.975		0.265		1.165
Han	485,192(99.0)	3364.998 ± 420.453		7069(1.5)		29,141(6.0)	
Other	4951 (1.0)	3359.067 ± 427.861		77(1.6)		316(6.4)	

*P < 0.05

### The relationship between maternal age and birth weight, LBW and macrosomia

**Fig. 2:**
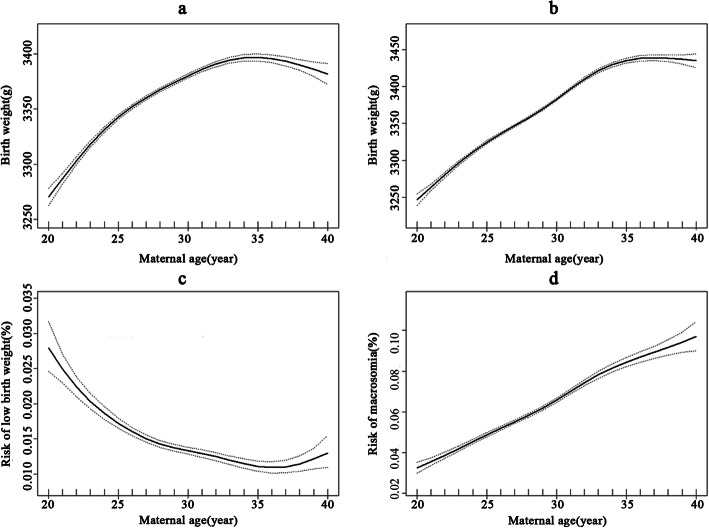
Relationships between maternal age and BW, LBW and macrosomia with 95% confidence interval (dashed lines) *. **a**: Unadjusted BW; **b**: Adjusted BW; **c**: LBW **d**: Macrosomia. *: Adjusted: **b**, **c** and **d** adjusting for gestational age, season of birth and maternal residence)

Both unadjusted and adjusted smoothed plots suggest that there was a nonlinear relationship between maternal age and BW (Fig. 2a, b). BW increased gradually until age 34, then decreased. Similarly, both adjusted smoothed plots suggest that there were nonlinear relationships between maternal age and LBW and macrosomia (Fig. 2c, d). The risk of LBW decreased gradually until age 36, then increased. The risk of macrosomia increased with the increase of maternal age when maternal age ranged from 20 to 40 years old.

Two turning points of maternal age for BW were found at 24 and 34 years old. (Table 2) The BW increased 16.204 g per year increase of maternal age up to the 24 years old (β = 16.204, 95%CI: 14.323, 18.086). The BW increased 12.051 g per year increase of maternal age when maternal age ranged from 24 to 34 years old (β = 12.051, 95%CI: 11.609, 12.493). But there was not significantly association between maternal age and BW when maternal age was older than 34 years old (β=-0.824, 95% CI: -3.112, 1.464).

**Table 2:** The effect of maternal age on BW in two-piece wise linear regression model

Turning point of maternal age	Maternal age	Adjusted *β/OR* (95%CI)^a^	*P* value
**Birth weight**			
24y,34y	20 ≤ and < 24 y	16.204 (14.323, 18.086)	< 0.001
	24 ≤ and ≤ 34 y	12.051 (11.609, 12.493)	< 0.001
	34 < and ≤ 40 y	-0.824 (-3.112, 1.464)	0.480
**Low birth weight**			
27y,36y	20 ≤ and < 27 y	0.917 (0.903, 0.932)	< 0.001
	27 ≤ and ≤ 36 y	0.965 (0.955, 0.976)	< 0.001
	36 < and ≤ 40 y	1.133 (1.026, 1.250)	0.013
**Macrosomia**			
24y,33y	20 ≤ and < 24 y	1.102 (1.075, 1.129)	< 0.001
	24 ≤ and ≤ 33 y	1.065 (1.060, 1.071)	< 0.001
	33 < and ≤ 40 y	1.029 (1.012, 1.046)	0.001

^a^Adjusted: adjusting for gestational age, season of birth, maternal residence

Two turning points of maternal age for LBW were found at 27 and 36 years old (Table 2). The risk of LBW decreased by 8.3% per year increase of maternal age up to 27 years old (Odds Ratio (OR) = 0.917, 95%CI: 0.903, 0.932). The risk of LBW decreased by 3.5% per year increase of maternal age when maternal age ranged from 27 to 36 years old (OR = 0.965, 95%CI: 0.955, 0.976). The risk of LBW increased by 13.3% per year increase in maternal age when maternal age was older than 36 years old (OR = 1.133, 95%CI: 1.026, 1.250). Similarly, two turning points value of maternal age for macrosomia were found at 24 and 33 years (Table 2). The risk of macrosomia increased by 10.2% per year increase of maternal age up to 24 years old (OR = 1.102, 95%CI: 1.075, 1.129). The risk of macrosomia increased by 6.5% per year increase of maternal age when maternal age ranged from 24 to 33 years old (OR = 1.065, 95%CI: 1.060, 1.071). The risk of macrosomia increased by 2.9% per year increase of maternal age when maternal age was older than 33 years old (OR = 1.029, 95%CI: 1.012, 1.046).

## Discussion

### Main results

Our research indicated the specific relationships between maternal age change and the change of BW, LBW and macrosomia. BW increased gradually until maternal age 34, then decreased. The risk of LBW decreased gradually until age 36, then increased. The risk of macrosomia increased with the increase of maternal age. The results of this study on BW showed that the relationship between the risk of abnormal BW and age is not based on the traditional 35-year-old boundary. Our findings provided the absolute risks of abnormal BW by maternal age, which would be useful for patient fertility counseling.

### Interpretation

A nonlinear relationship between maternal age and BW was observed and two turning points of maternal age at 24 and 34 years old were found in this study. The BW increased faster from 20 to 23 years old than from 24 to 34 years old. The curve was consistent with the findings of the previous studies [12, 13], however, there was no research provided that the threshold maternal age on BW was 34 years old [12, 21, 22]. We observed a marginal significantly negative association between maternal age and BW when maternal age ranged from 35 to 40 years, which was consistent with the findings of the previous studies [23, 24]. In previous studies, the population was grouped according to maternal age into several subgroups, then to investigate the effect of maternal age on the BW. Most studies considered women in age group 20–29 years as reference group. Differently, in our study, the maternal age was analyzed as a continuous variable rather than categorical variable, to find a more accurate age threshold.

The mechanism of the effect of maternal age on BW is still unclear. In the study, we found the risks of LBW and macrosomia after 35 years present the same pattern, increasing at these maternal age period. Relevant researches showed that most of the effects on the offspring of intrauterine exposure to maternal age-related obstetric complications might be induced by epigenetic DNA reprogramming during critical periods of the embryo or fetal development [25]. And it is well known that mitochondria are maternally derived. We also know that mitochondrial DNA is not capable of DNA repair and is therefore at greater risk of acquiring mutations with age.

[12]. Similarly, the quality of woman’s eggs declined dramatically with increasing age, leading to an increased risk of pregnancy-related complications among older women [12]. Furthermore, older mothers are more likely to suffer from chronic diseases and their complications in pregnancy, including obesity, anemia and diabetes. In addition, ageing process affects the reproductive system similar to other systems in the human body. These factors may contribute to that the risks of LBW and macrosomia after 35 years present the same pattern, increasing at these maternal age period.

A nonlinear relationship between maternal age and the risk of term LBW was found in our study. The curve was the same as the previous study [13, 26]. As LBW newborns may be premature with other risk factors [27], we restricted our study population to term newborns, therefore the etiology of LBW was intrauterine growth restriction [18]. The prevalence of term LBW in our study was 1.5%, which was lower than 2% reported by the other study [28], which suggesting there was a good perinatal care system in Xi’an, China. Although the prevalence of term LBW is low, it is not negligible and term LBW can lead to adverse pregnancy outcomes, as severe neonatal asphyxia [29]. The threshold maternal age on the risk of term LBW was 36 years old, which means that the risk of term LBW will significantly increase when the maternal age is over 36 years old, but fewer studies have reported it [5, 6, 14]. However, the biological mechanisms by which maternal age cause the term LBW are unclear. The increased risk of LBW among younger can be explained by the low socioeconomic conditions and increased nutritional demands of pregnancy [30].

With the increase of maternal age, the risk of macrosomia was increasing and two turning points of maternal age were found at 24 and 33 years. The curve was identical to the previous studies [31]. The incidence of macrosomia was 6.0% in our study, which was approximate with the previous study [14]. The risk of macrosomia increased faster from 20 to 23 years old than from 24 to 33 years old, which was the same as the change of BW. Term macrosomia is influenced by maternal hyperglycemia and endocrine status through placental circulation [32]. The increased risk of term macrosomia is partly explained by the increased prevalence of diabetes with the increased maternal age [13].

### Strengths and limitations

Our research has some advantages: first, our study was a large sample research based on four years records. Besides, our study had very strict inclusion and exclusion criteria and the data was also carried on the strict cleaning and so on, which increases the credibility of the results. However, there were some potential limitations in this study. First, some potential confounders, such as fetus gender, parity, medical history, economic condition and paternal age, were not adjusted because of limited data. Although the cutoff for “paternal advanced age” is not clearly defined, there is an increase in genetic risk as men age. Previous studies showed that economic condition and fetus gender were associated with higher BW, but adjustment for socioeconomic factors and fetus gender made little difference to the results [22]. In addition, at any maternal age, higher parity was associated with higher BW [22].

Therefore, the extrapolation of the results requires caution, especially for others ethnicity [21]. However, the results of this study still have a certain degree of reference significance for other regions. Second, the mother-child pairs were not included when the maternal age was younger than 20 years old or older than 40 years old, because of the small proportion of them and limited data. Women delivering before 20 or after age 40 years had a higher incidence of pregnancy complications. Therefore, our research could only reflect the changing trend of BW, LBW and macrosomia when the maternal age ranged from 20 to 40 years old. In order to ensure the accuracy of our research results, we limited the subjects to full term singleton live birth, which could eliminate some potential influencing factors [27], such as serious pregnancy complications. Although the large sample size might increase potential confounding, we could estimate the relationships between maternal age and BW, LBW and macrosomia in detail with generalized additive model and two-piece wise linear regression model.

## Conclusions

Our research indicated the specific relationships between maternal age change and the change of BW, LBW and macrosomia when maternal age ranged from 20 to 40 years old. BW increased gradually until 34 years old, then decreased. The threshold maternal age for LBW was 36 years old, and the risk of macrosomia was increasing with the increase of maternal age. These results should be carefully taken into account by maternal care providers in order to inform women adequately, support them in understanding potential risks of BW associated with their age, and the importance of prenatal care. However, optimized maternal age should be determined individually by different pregnancy complications and adverse neonatal outcomes. The mechanism between maternal age and BW is not clear. Therefore, further studies are needed to examine the relationship between maternal age and BW.

## Data Availability

The datasets used and/or analyzed during the current study are available from the corresponding author on reasonable request.

## Abbreviations

BW: Birth weight
LBW: Low birth weight
β: Adjusted Effect size
OR: Odds Ratio
CI: Confidence intervals
